# Impact of India’s National Tobacco Control Programme on bidi and cigarette consumption: a difference-in-differences analysis

**DOI:** 10.1136/tobaccocontrol-2018-054621

**Published:** 2018-12-15

**Authors:** Gaurang P Nazar, Kiara C-M Chang, Swati Srivastava, Neil Pearce, Anup Karan, Christopher Millett

**Affiliations:** 1 Health Promotion Division, Public Health Foundation of India, Gurugram, India; 2 Department of Non-communicable Disease Epidemiology, London School of Hygiene and Tropical Medicine (LSHTM), London, UK; 3 Public Health Policy Evaluation Unit, School of Public Health, Imperial College London, London, UK; 4 Department of International Health, Johns Hopkins University Bloomberg School of Public Health, Baltimore, USA; 5 Indian Institute of Public Health, Delhi (IIPHD), Public Health Foundation of India, Gurugram, India; 6 Center for Epidemiological Studies in Health and Nutrition, University of São Paulo, São Paulo, Brazil

**Keywords:** non-cigarette tobacco products, low/middle income country, cessation, public policy, harm reduction

## Abstract

**Background:**

Despite the importance of decreasing tobacco use to achieve mortality reduction targets of the Sustainable Development Goals in low-income and middle-income countries (LMICs), evaluations of tobacco control programmes in these settings are scarce. We assessed the impacts of India’s National Tobacco Control Programme (NTCP), as implemented in 42 districts during 2007–2009, on household-reported consumption of bidis and cigarettes.

**Methods:**

Secondary analysis of cross-sectional data from nationally representative Household Consumer Expenditure Surveys (1999–2000; 2004–2005 and 2011–2012). Outcomes were: any bidi/cigarette consumption in the household and monthly consumption of bidi/cigarette sticks per person. A difference-in-differences two-part model was used to compare changes in bidi/cigarette consumption between NTCP intervention and control districts, adjusting for sociodemographic characteristics and time-based heterogeneity.

**Findings:**

There was an overall decline in household-reported bidi and cigarette consumption between 1999–2000 and 2011–2012. However, compared with control districts, NTCP districts had no significantly different reductions in the proportions of households reporting bidi (adjusted OR (AOR): 1.03, 95% CI: 0.84 to 1.28) or cigarette (AOR: 1.01 to 95% CI: 0.82 to 1.26) consumption, or for the monthly per person consumption of bidi (adjusted coefficient: 0.07, 95% CI: −0.13 to 0.28) or cigarette (adjusted coefficient: −0.002, 95% CI: −0.26 to 0.26) sticks among bidi/cigarette consuming households.

**Interpretation:**

Our findings indicate that early implementation of the NTCP may not have produced reductions in tobacco use reflecting generally poor performance against the Framework Convention for Tobacco Control objectives in India. This study highlights the importance of strengthening the implementation and enforcement of tobacco control policies in LMICs to achieve national and international child health and premature NCD mortality reduction targets.

**What this paper adds**

- Evaluations of tobacco control programmes in low-income and middle-income countries (LMICs) are scarce but essential given the importance of reducing tobacco use to achieve both child and adult mortality reduction targets in the Sustainable Development Goals.
- This first evaluation of India’s National Tobacco Control Programme (NTCP) (implemented during 2007–2009) found that although India’s overall consumption of bidis and cigarettes declined between 1999–2000 and 2011–2012, the observed reductions were not significantly different between NTCP and non-NTCP districts.
- Our findings indicate that early implementation of the NTCP may not have produced reductions in tobacco use. This may reflect inadequacies in initial programme implementation and highlight the need to strengthen operationalisation of tobacco control programmes in India and other LMICs.

## Introduction

India is home to 267 million tobacco users,^1^ the second largest number of tobacco consumers in the world and the country faces a substantial tobacco-related mortality and morbidity burden.^2 3^ Efforts to strengthen tobacco control in India are underpinned by the enactment of the Cigarettes and Other Tobacco Products Act (COTPA) in 2003 and its ratification of the WHO Framework Convention on Tobacco Control (FCTC) in 2004.^4 5^ India’s commitment towards the provision, and more effective implementation and enforcement of tobacco control measures under COTPA and FCTC led to the introduction of the National Tobacco Control Programme (NTCP) in 2007–2008.^6^ Initially developed as a pilot project in two districts in each of nine Indian states,^7^ the NTCP was expanded in 2008–2009 to cover a total of 42 districts and 21 states (online supplementary table S1) and has been expanded to 400 districts across India with a budget allocation of INR 650 million (US$8.8 million) for the year 2018–2019.^8^

10.1136/tobaccocontrol-2018-054621.supp1 Supplementary file 1

Key objectives of the NTCP are to: (a) increase awareness about existing tobacco control laws and the harmful effects of tobacco use; and (b) facilitate effective implementation of tobacco control laws and policies. NTCP activities include district level establishment and expansion of tobacco cessation facilities, school-based tobacco control programmes, training and capacity building for teachers, health professionals and other stakeholders, and monitoring of tobacco control activities under COTPA.^6 7^ While both national-level and state-level governments have an important role in tobacco control including taxation policy, public awareness campaigns, establishment of tobacco testing laboratories and research into alternative crops, much of the focus of NTCP activities is at the district level.

Robust assessment of tobacco control programmes in low-income and middle-income countries (LMICs) is essential given the importance of reducing tobacco use to achieve both child and adult mortality reduction targets in the Sustainable Development Goals (SDGs).^9 10^ This includes regular monitoring of achievement against programme goals and objectives and ensuring that resources are allocated properly and spent effectively.^11^ However, robust monitoring and evaluation in LMICs is often constrained by lack of surveillance data although new initiatives are seeking to address this, such as the International Tobacco Control Policy Evaluation Project for India which aims to collect longitudinal survey data on key FCTC policy measures from around 10 600 adult informants in four states.^12^ Its wave 1 (2010–2011) findings have highlighted a slow and inconsistent progression of tobacco control policies across states (eg, pictorial health warnings, smoke-free public places) and an urgent need to strengthen them.^13^

Since the introduction of the NTCP, operational challenges have been reported including insufficient staffing and mechanisms to monitor the programme, and an evaluation of programme effectiveness is not available to date.^4–6^ Therefore, we aimed to evaluate impacts of the early phase of the NTCP implemented during 2007–2009, on household level bidi (Bidi is made by rolling a dried, rectangular piece of temburni leaf (Diospyros melanoxylon) with 0.15–0.25 g of sun-dried, flaked tobacco into a conical shape and securing the roll with a thread.) and cigarette consumption using large and nationally representative Consumer Expenditure Surveys (CESs) of India.

## Methods

### Study design, setting and data

We used the three most recent waves (1999–2000, 2004–2005 and 2011–2012) of the nationally representative, household CES data conducted by the National Sample Survey Organization, Government of India.^14–16^ The CES used a stratified multistage sampling design covering districts from all states and union territories in India. The head of household or equivalent (adult participant aged ≥15 years) of randomly selected households were invited to participate in a face-to-face interview, and a validated interviewer-administered questionnaire was used to obtain information about the household’s consumption and expenditure of over 350 food and non-food items. Full details of the CES are available elsewhere.^14–16^

The sample sizes of the three survey waves varied between 100 000 and 125 000 households and spread across approximately 12 000 subdistricts (villages or urban blocks).^14–16^ These sum up to 341 975 households included in our study after excluding 4640 households (1.3% of 346 615 households) that had no or incomplete data recorded.

### Outcome measures

We considered four different outcomes: (a) proportion of households reporting consumption of bidis, (b) proportion of households reporting consumption of cigarettes, (c) number of bidi sticks consumed per person in households reporting bidi use and (d) number of cigarette sticks consumed per person in households reporting cigarette use. These were all based on reported consumption in the 30 days before the interview.

### Explanatory variables

Main explanatory variables were: (a) an NTCP indicator, equals to 1 for households residing in an NTCP district (those listed in the operational guidelines of NTCP, and online supplementary table S1), 0 otherwise^7^; (b) two time indicators, t_2_ (1 for survey year 2004–2005, 0 otherwise) and t_3_ (1 for survey year 2011–2012, 0 otherwise); and (c) two interaction terms: (1) an interaction term between NTCP indicator and t_2_ (DID1); and (2) an interaction term between the NTCP indicator and t_3_ (DID2).

We controlled for an array of demographic and socioeconomic status (SES) that have been previously shown associated with tobacco smoking in India.^17 18^ These included household’s area of residence (rural or urban) and wealth quintile (poorest, poor, middle, rich, richest); household size (number of household members ≤5 or >5); proportion of household members in each age group (0–4 years; 5–14 years; 15–29 years; 30–59 years; ≥60 years); proportion of male and female members in the household, proportion of household members at each educational level (illiterate, primary, middle, secondary, higher secondary, graduate and above); household religion (Hindu, Muslim, Christian and others); caste (A system of rigid social stratification characterised by hereditary status, endogamy, and social barriers sanctioned by custom, law or religion.) (scheduled tribe, scheduled caste, other backward class and others); and employment type (self-employed, regular labourer, casual labourer and others).^19^ Household characteristics of all three survey years are presented in online supplementary table S2.

### Statistical analysis

We employed a quasi-experimental difference-in-differences (DID) analysis to assess impacts of the NTCP on household-reported consumption of bidis and cigarettes. DID analysis is an important and commonly used method for policy impact evaluations.^20–22^ It usually involves intervention and control groups observed at two time periods while only the intervention group is affected by the policy at the second time period. The DID model estimates policy effect by comparing the average changes in outcome before and after the introduction of the policy among the intervention group, and subtracting from it, the average changes in outcome over the same time period among the control group.

An unbiased DID estimator must fulfil the assumption of parallel trend which requires that in the absence of the policy, the outcomes of the intervention and control groups will follow a parallel trend over time—a property that cannot be observed since the policy is already implemented.^20^ Therefore, in this study, we included data collected for two time periods before (1999–2000 and 2004–2005) and one time period after (2011–2012) the introduction of the NTCP (2007–2009). Our DID analyses involved all main explanatory variables as defined above. The NTCP indicator captures the difference in outcome between intervention and control groups at baseline (1999–2000). The time indicator t_2_ captures the slope changes in outcome between baseline and year 2004–2005 for the control group whereas the interaction term, DID1, assesses a departure from this slope for the intervention group. The latter is also the test of parallel trend assumption prior to NTCP implementation, and we defined a statistically significant DID1 at 5% as potential violation of assumption.

The time indicator t_3_ captures the slope changes in outcome between baseline and year 2011–2012 for the control group whereas the interaction term, DID2, assesses a departure from this slope for the intervention group. The statistical difference between the two interactions terms, DID1 and DID2, therefore captures the changes in outcome that may be attributable to the NTCP.^21^

Since the quantity of household bidi and cigarette consumption is always positive although left-skewed with a large number of zeros, we embedded the proposed DID specification into a two-part model,^21 23 24^ where part 1 uses a logit model to estimate the prevalence of households reporting consumption of bidis/cigarettes, part 2 uses a linear model to estimate the log-transformed monthly number of bidis/cigarettes sticks consumed per person, conditional on the households reporting any bidi/cigarette smoking. Please refer to online supplementary file 1 for detailed specification of the DID within two-part model framework.

Presence of any contamination and/or spill-over effects may result in underestimated programme impacts if NTCP activities influenced neighbouring non-NTCP districts. Therefore, we ran the same statistical model on three different control groups for comparison: (a) model 1 included controls from both NTCP and non-NTCP states, (b) model 2 included controls from NTCP states only and (c) model 3 included controls from non-NTCP states only. We adjusted in the regression models household demographic and SES indicators, and state level fixed effects. We used robust standard errors to account for clustering at district level, and CES-provided sampling weights were applied. For comparison purpose, we additionally ran a combined DID and matching analysis which used 1:1 nearest neighbour matching without replacement to find for each NTCP district, a propensity score matched non-NTCP district based on district level sociodemographic characteristics prior to programme introduction.^20^ All statistical analyses were conducted using STATA V.13.1 (StataCorp).

## Results

### Descriptive statistics

**Figure 1:**
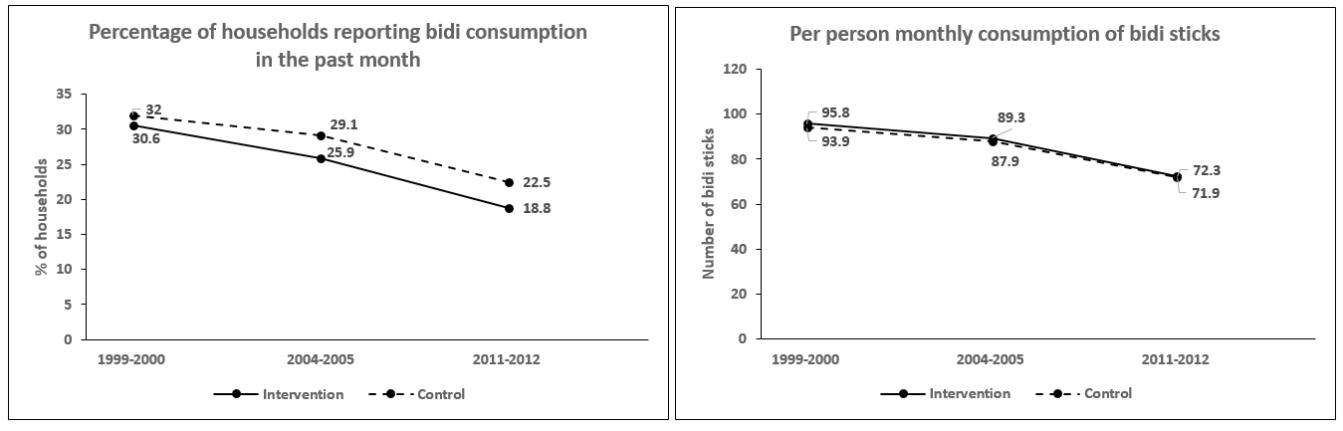
Unadjusted household consumption of bidi over Consumer Expenditure Survey rounds (1999–2000; 2004–2005; 2011–2012) in National Tobacco Control Programme (NTCP) districts versus non-NTCP districts.

**Figure 2:**
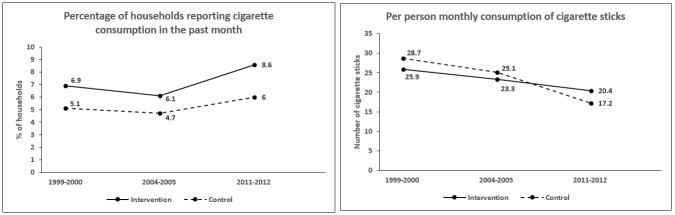
Unadjusted household consumption of cigarette over Consumer Expenditure Survey rounds (1999–2000; 2004–2005; 2011–2012) in National Tobacco Control Programme (NTCP) districts versus non-NTCP districts.

The unadjusted prevalence of households-reported bidi consumption declined from 31.9% to 28.8%, and 22.1% across the three survey waves whereas prevalence of cigarette consumption increased slightly from 5.3% to 4.8%, and 6.3%, respectively. These patterns were similar in households located in NTCP and non-NTCP districts (figures 1 and 2).

The unadjusted monthly consumption of bidis declined from 94.0 to 88.0, and 71.9 sticks per person over the study period whereas the monthly consumption of cigarettes declined from 28.4 to 24.8, and 17.6 sticks per person. Reductions in the consumption of bidi sticks were almost identical between households residing in the NTCP versus non-NTCP districts (figure 1). However, the consumption of cigarette sticks appeared to decline more sharply between 2004–2005 and 2011–2012 among households residing in non-NTCP districts than those in NTCP districts (figure 2).

### Impact of the NTCP on bidi consumption

We present in table 1 adjusted results for the changes in prevalence of household-reported bidi consumption over time in part 1, and the changes in log-transformed monthly consumption of bidi sticks per person in part 2.

**Table 1:** Effects of India’s National Tobacco Control Programme (NTCP) on bidi consumption (Consumer Expenditure Survey 1999–2000; 2004–2005; 2011–2012 pooled data)

Part 1: Prevalence of households reporting bidi consumption, adjusted OR (95% CI)			
	Model 1	Model 2	Model 3
	n=341 975	n=270 265	n=107 291
Constant	0.49 (0.15 to 1.55)	0.39* (0.31 to 0.50)	0.59* (0.20 to 1.74)
NTCP indicator	0.99 (0.80 to 1.23)	1.00 (0.81 to 1.24)	0.44 (0.16 to 1.21)
Time indicator, t_2_	0.70* (0.65 to 0.74)	0.71* (0.67 to 0.76)	0.55* (0.49 to 0.63)
Time indicator, t_3_	0.74* (0.69 to 0.79)	0.76* (0.70 to 0.81)	0.62* (0.53 to 0.73)
Interaction term, DID1	0.99 (0.77 to 1.26)	0.96 (0.75 to 1.23)	1.21 (0.93 to 1.58)
Interaction term, DID2	1.03 (0.78 to 1.35)	1.01 (0.76 to 1.33)	1.18 (0.88 to 1.57)
Effects attributable to NTCP	1.03 (0.84 to 1.28)	1.04 (0.84 to 1.30)	0.97 (0.75 to 1.24)

Part 2: Log-transformed monthly consumption of bidi sticks per person, conditional on the households reporting bidi consumption, adjusted coefficient (95% CI)			
	Model 1	Model 2	Model 3
	**n=**86 818	**n=**69 366	**n=**25 767
Constant	4.24* (4.09 to 4.39)	3.24* (3.10 to 3.38)	4.17* (3.96 to 4.38)
NTCP indicator	0.03 (-0.10 to 0.16)	0.03 (-0.10 to 0.16)	−0.24* (−0.46 to -0.01)
Time indicator, t_2_	−0.11* (−0.16 to -0.07)	−0.11* (−0.16 to -0.06)	−0.15* (−0.24 to -0.05)
Time indicator, t_3_	−0.37* (−0.44 to -0.31)	−0.36* (−0.43 to -0.29)	−0.51* (−0.62 to -0.40)
Interaction term, DID1	−0.04 (-0.23 to 0.13)	−0.05 (-0.23 to 0.13)	0.002 (-0.19 to 0.19)
Interaction term, DID2	0.02 (-0.13 to 0.19)	0.02 (-0.14 to 0.19)	0.10 (-0.09 to 0.30)
Effects attributable to NTCP	0.07 (-0.13 to 0.28)	0.07 (-0.13 to 0.28)	0.10 (-0.14 to 0.34)

Explanation of variables:

NTCP indicator equals to 1 for households residing in an NTCP district, 0 otherwise.

t_2_ equals to 1 for survey year 2004–2005, 0 otherwise.

t_3_ equals to 1 for survey year 2011–2012, 0 otherwise.

DID1 was the interaction between NTCP indicator and t_2._

DID2 was the interaction between NTCP indicator and t_3._

Effects attributable to NTCP was calculated as the difference of DID2–DID1.

These three different types of control groups were modelled against households residing in an NTCP district.

Model 1: control group included households residing in a non-NTCP district from any state.

Model 2: control group included households residing in a non-NTCP district situated in a state where some districts were NTCP-implemented sites.

Model 3: control group included households residing in a non-NTCP district located in a state with no NTCP activities in any of its districts.

All regression models were adjusted for demographic and socioeconomic status of the households including sector (rural/urban); size (≤5/ >5 members); proportion of members in each age-group (0–4, 5–14, 15–29, 30–59, ≥60); proportion of females/males; proportion of members in each educational level (illiterate, primary, middle, secondary, higher secondary, graduate and above); religion (Hindu/Muslim/Christian/others); caste (scheduled tribe/scheduled caste/other backward class/others); employment type (self-employed/regular labour/casual labour/others); and wealth quintile (poorest/poor/middle/rich/richest); and state level fixed effects.

*P<0.05.

DID, difference-in-differences.

Based on the findings for model 1, both the prevalence of bidi consumption and the log-transformed bidi sticks consumed monthly per person declined significantly between 1999–2000 and 2004–2005, and between 2004–2005 and 2011–2012. The test of parallel trend assumption between the first two time periods prior to NTCP introduction showed no evidence of a violation for both outcomes (eg, adjusted OR (AOR) for DID1 in part 1 was 0.99 (95% CI: 0.77 to 1.26)).

The NTCP was not found to have a significantly different impact in intervention compared with control districts in model 1; AOR of 1.03 (0.84 to 1.28) for bidi smoking prevalence and an adjusted coefficient of 0.07 (−0.13 to 0.28) bidi sticks per month per person. model 2 and model 3 assessed the possibilities of contamination and/or spill-over effects influencing model 1 findings, but their results were consistent with model 1 for both outcomes. Furthermore, results based on propensity score matched intervention and control groups were largely similar to model 1 (online supplementary table S3).

### Impact of the NTCP on cigarette consumption

Model 1 findings suggest that after accounting for all explanatory variables in the regression models, prevalence of cigarette consumption did not change significantly over the study period although the log-transformed cigarette sticks consumed monthly per person declined significantly (table 2). The test of parallel trend assumption (DID1) between the first two time periods prior to NTCP introduction again showed no evidence of a violation for both outcomes (eg, AOR for DID1 in part 1 was 0.85 (0.59 to 1.21)).

**Table 2:** Effects of India’s National Tobacco Control Programme (NTCP) on cigarette consumption (Consumer Expenditure Survey 1999–2000; 2004–2005; 2011–2012 pooled data)

Part 1: Prevalence of households reporting cigarette consumption, adjusted OR (95% CI)			
	Model 1	Model 2	Model 3
	n=341 975	n=270 265	n=107 291
Constant	0.07* (0.04 to 0.10)	0.03* (0.03 to 0.04)	0.10* (0.05 to 0.19)
NTCP indicator	1.23 (0.89 to 1.70)	1.21 (0.87 to 1.67)	0.46* (0.32 to 0.66)
Time indicator, t_2_	1.01 (0.92 to 1.11)	0.95 (0.85 to 1.06)	1.29* (1.11 to 1.49)
Time indicator, t_3_	1.18* (1.06 to 1.31)	1.18* (1.05 to 1.34)	1.20 (0.99 to 1.45)
Interaction term, DID1	0.85 (0.59 to 1.21)	0.89 (0.62 to 1.28)	0.73 (0.51 to 1.05)
Interaction term, DID2	0.86 (0.59 to 1.26)	0.85 (0.58 to 1.25)	0.97 (0.64 to 1.45)
Effects attributable to NTCP	1.01 (0.81 to 1.26)	0.95 (0.76 to 1.19)	1.32* (1.00 to 1.73)

Part 2: Log-transformed monthly consumption of cigarette sticks per person conditional on the households reporting cigarette consumption, adjusted coefficient (95% CI)			
	Model 1	Model 2	Model 3
	n=86 818	n=69 366	n=25 767
Constant	2.54* (2.14 to 2.94)	1.12* (0.47 to 1.77)	3.02* (2.50 to 3.54)
NTCP indicator	−0.19 (-0.40 to 0.02)	−0.21 (-0.40 to 0.002)	−1.89* (−2.38 to 1.39)
Time indicator, t_2_	−0.14* (−0.23 to -0.06)	−0.16* (−0.26 to -0.06)	−0.06 (-0.21 to 0.09)
Time indicator, t_3_	−0.51* (−0.61 to -0.41)	−0.57* (−0.68 to -0.47)	−0.30* (−0.50 to -0.09)
Interaction term, DID1	0.19 (−0.06 to 0.45)	0.19 (−0.06 to 0.45)	0.09 (−0.18 to 0.36)
Interaction term, DID2	0.19 (−0.08 to 0.46)	0.24 (−0.02 to 0.51)	−0.08 (−0.40 to 0.24)
Effects attributable to NTCP	−0.002 (−0.26 to 0.26)	0.04 (−0.22 to 0.31)	−0.17 (−0.46 to 0.11)

Explanation of variables:

NTCP indicator equals to 1 for households residing in an NTCP district, 0 otherwise.

t_2_ equals to 1 for survey year 2004–2005, 0 otherwise.

t_3_ equals to 1 for survey year 2011–2012, 0 otherwise.

DID1 is the interaction between NTCP indicator and t2.

DID2 is the interaction between NTCP indicator and t3.

Effects attributable to NTCP was calculated as the difference of DID2–DID1.

These three different types of control group were modelled against households residing in an NTCP district:

Model 1: control group included households residing in a non-NTCP district from any state.

Model 2: control group included households residing in a non-NTCP district situated in a state where some districts were NTCP-implemented sites.

Model 3: control group included households residing in a non-NTCP district located in a state with no NTCP activities in any of its districts.

All regression models were adjusted for demographic and socioeconomic status of the households including sector (rural/urban); size (≤5/ >5 members); proportion of members in each age-group (0–4, 5–14, 15–29, 30–59, ≥60); proportion of females/males; proportion of members in each educational level (illiterate, primary, middle, secondary, higher secondary, graduate and above); religion (Hindu/Muslim/Christian/others); caste (scheduled tribe/scheduled caste/other backward class/others); employment type (self-employed/regular labour/casual labour/others); and wealth quintile (poorest/poor/middle/rich/richest); and state level fixed effects.

*P<0.05.

The NTCP was not found to have a significantly different impact in intervention compared with control districts for cigarette smoking prevalence (AOR: 1.01 (0.81 to 1.26)), or for log-transformed cigarettes sticks consumed monthly per person (adjusted coefficient: −0.002 (−0.26 to 0.26)). Results were largely similar for model 2 and model 3.

## Discussion

Our findings suggest that between 1999–2000 and 2011–2012, there was an overall reduction in the household consumption of bidis and cigarettes in India. However, our adjusted analyses found no evidence that the observed reductions in bidi or cigarette consumption were significantly different between NTCP and non-NTCP districts, 3–4 years after programme implementation. These findings are important as they add to the scant evidence base concerning whether tobacco control programmes are optimally designed and implemented in LMICs such as India.^12^

Our findings may reflect the substantial challenges and operational obstacles encountered during early stages of the NTCP, these include: insufficient staffing, resource allocation and utilisation, and a lack of effective mechanisms for monitoring and ensuring compliance with the programme.^4 6^ By 2012, published estimates have suggested that among 21 NTCP states, only 50% had mechanisms in place to monitor compliance of tobacco control interventions; 50% collected penalties for the violation of smoke-free law at public places; 14% collected penalties for the violation of ban on tobacco advertising; compliance with the ban on sale of tobacco products to and by minors, and ban on sale within 100 yards of educational institutions remained poor in many of the states; and smoking cessation facilities were absent from districts in almost 50% of the states.^4 25^ Two recent cross-sectional studies based in Delhi, India, have both highlighted the unsatisfactory compliance with COTPA due to the absence of a display board outside educational institutions stating prohibition of sale of tobacco products within a radius of 100 yards, and the high frequency of sale of tobacco products within 100 yards of educational institutions.^25 26^

The overall decline in bidi and cigarette use (in both NTCP and non-NTCP districts) identified here are consistent with more recent data from India’s 2016–2017 Global Adult Tobacco Survey (GATS).^1^ This shows that bidi use declined by 16.3% (from 9.2% to 7.7%) and that cigarette use declined by 29.8% (from 5.7 to 4.0) between 2009–2010 and 2016–2017 among adult participants aged 15 years or above.^1^ Our findings provide grounds to challenge the assumption that this decline is attributable to the NTCP. Our assertion is supported by more recent evidence indicating that key tobacco control measures adopted by the NTCP under COTPA,^27^ including smoke-free laws, pictorial health warnings and banning advertising and promotion, have been poorly implemented and far from meeting international best practices. Self-reported exposure to secondhand smoke at work has not decreased in the past 7 years (30.0% in 2009–2010 and 30.2% in 2016–2017 GATS report).^1^ The implementation of mandatory pictorial health warnings has been weak and substantially delayed.^28^ Warning labels covering only 40% on front panel of tobacco packets were introduced in 2009 with implementation of WHO recommended labels which covers 85% on both sides of the packet being delayed until 2016.^27 28^ While all forms of tobacco advertising (except at point of sale and on tobacco packages) were prohibited in 2004 when COTPA came into force, more than one in five adults in India are still exposed to advertising and promotion of tobacco products as shown in the 2016–2017 GATS report.^1^ The declines in tobacco use seen may be due to other factors, such as increased awareness about the harms of smoking and tobacco chewing. Modest investment in tobacco control activities under COTPA and national mass media campaigns which were part of the NTCP but not evaluated here may have contributed to this decline.

Poor implementation of the NTCP should be viewed in the context of wider weaknesses in the implementation of tobacco control laws in India. For example, while the NTCP does not have responsibility for taxation of tobacco products, tax remains an underused tool to reduce tobacco consumption in India.^27^ In 2016, taxes on bidis and cigarettes were well below WHO recommended levels at 19.5% and 43.1%, respectively, with substantial differences in taxes levied between products; and the disparities in tax exemptions/subsidies remain an alarming issue in India, such as the tax exemption granted for manufacturers of hand-made bidis and those with a turnover below certain threshold.^29 30^ The Indian government has sought to standardise tobacco tax policy with the introduction of a national Good and Services Tax in July 2017; however, its impact awaits future evaluation.

Tobacco control programmes in high-income countries have shown that a comprehensive, well-resourced and aggressively implemented strategy is required to effectively reduce tobacco consumption.^31–34^ These tobacco control programmes share many similarities to the NTCP, such as introducing and implementing laws on smoke-free public places, mass media campaigns, school-based interventions, strengthening tobacco cessation services and continuously monitoring adherence to tobacco control policies. However, these programmes had additionally introduced raised taxation on tobacco products, were well financed and managed, and had been supported by regular programme evaluations and monitoring against programme objectives. Comparison of the programme funding shows that the 2018/2019 budget allocated to the NTCP equates to US$0.009 per capita (US$8.8 million/911.6 million adults aged ≥15)^8 35^ which is two times above the WHO estimated tobacco control expenditure among low-income countries (US$0.0004), but this is only one-third of the amount spent by middle-income countries (US$0.03 per capita) and below 1% of those spent by high-income countries (US$1.26 per capita).^36^ Furthermore, the consumption of cigarettes per capita dropped by 12% following the implementation of Massachusetts’ comprehensive tobacco control programme and 4% annually thereafter.^34^ California’s comprehensive tobacco control programme implemented in 1989 was associated with 1.52 times faster decline in cigarette consumption per capita during 1989–1993.^32 33^ However, the rate of decline slowed during 1994–1996, probably due to a combination of reduced programme funding and aggressive tobacco industry tactics.^32^ The association between expenditures on tobacco control programmes and reduction in smoking prevalence has been demonstrated in a US study, indicating that doubling of funding can potentially reduce smoking prevalence by 1% to 1.7%.^37^ Robust evaluation of tobacco control programmes in LMIC settings is sparse. One exception is Brazil which has implemented and evaluated a set of ambitious tobacco control policies that have been associated with a 50% reduction in smoking prevalence over the past two decades.^38^

Given this context and the growing burden of tobacco smoking in India,^39^ the suboptimal impacts of the NTCP on bidi and cigarette consumption and associated forgone health benefits is concerning.^1^ Modelling studies have shown that a comprehensive and effectively implemented tobacco control strategy for India is expected to avert four million myocardial infarctions and stroke deaths over the next decade.^40^ Recent evaluations on England’s smoke-free legislation have repeatedly shown positive impacts, including reduced infant deaths and other adverse birth outcomes, and reduced hospital admissions for childhood asthma and respiratory tract infections.^41–43^ These findings highlight the importance of strengthening tobacco control interventions and policies in India and other LMIC to achieve SDG targets for improving child health and reducing premature mortality from non-communicable diseases.^9 10^

### Strengths and limitations

This study is the first to quantify impacts of India’s NTCP on household consumption of bidis and cigarettes using large and nationally representative, repeated cross-sectional surveys of India. In the absence of gold-standard randomised controlled trials, the quasi-experimental DID design attempts to mimic a trial design by comparing intervention and control groups and changes in outcomes before and after policy implementation. The DID estimator is based on an assumption of parallel trends which our analyses of preintervention time trends suggest have not been violated. Furthermore, we considered the possibility that estimates of programme impacts are conservative due to contamination and/or spill-over effects of the NTCP activities influencing non-NTCP districts. However, our additional analyses showed no evidence for the presence of such biases.

Our study has a number of limitations. First, it was not possible to adjust for other tobacco control activities administered locally, outside of NTCP’s remit, although we were not aware of any. Moreover, due to the challenges encountered during early stages of the NTCP programme, the intensity of programme implementation may have varied across states and districts. Second, although the main focus of the NTCP was to improve and strengthen local tobacco control interventions at district level, some national and state level activities were undertaken. Our model 3 compared households in NTCP districts (and therefore located within NTCP states) with households in a non-NTCP district located within non-NTCP states. The null findings for model 3 suggest that state level activities do not have a discernible impact on bidi and cigarette consumption. Third, we did not include smokeless tobacco in this study because regulations for smokeless tobacco products were managed separately in India, for example, through the Food Safety and Standards (Prohibition and Restriction on Sales) Regulations.^44^ Also, the CES collected data for a variety of smokeless tobacco products, but they were recorded in different units and not all products were captured. Fourth, the self-reported nature of the CES data may be subject to social desirability bias. Fifth, we assessed NTCP impacts 3–4 years following programme implementation and longer follow-up time may be required for programme benefits to be realised. Moreover, the NTCP may be better placed in an integrated system following India’s recent transition towards Comprehensive Primary Health Care which aims to move primary healthcare centres closer to the local population and to ensure better access to a wider range of preventive, promotive and curative services instead of the former system that only focused on pregnancy, child health and few other disease conditions.^45^ It is crucially important to continue evaluating and monitoring changes in tobacco consumption in India, as further expansion of the NTCP to all other districts is currently under way.

## Conclusions

Although India’s overall consumption of bidis and cigarettes declined between 1999–2000 and 2011–2012, these observed reductions were not significantly different between NTCP and non-NTCP districts. These findings highlight the importance of strengthening the implementation and enforcement of tobacco control policies in LMICs to achieve SDG mortality reduction targets for children and adults.
