# Supplementary material for: Impact of India’s National Tobacco Control Programme on bidi and cigarette consumption: a difference-in-differences analysis
Source: Tob Control. 2018 Dec 15;29(1):103–10. doi: 10.1136/tobaccocontrol-2018-054621 (PMC6952846; doi:10.1136/tobaccocontrol-2018-054621)
Supplement: Supplementary file 1 [file tobaccocontrol-2018-054621supp001.pdf]

APPENDIX

Supplementary Table S1: Name of the two districts in each of the 21 states of India where the National Tobacco Control Programme was implemented between 2007-2009

	Name of State	Name of Districts
**Phase 1 (2007-2008)**		
**1**	Assam	Kamrup, Johrat
**2**	Delhi	New Delhi, East Delhi
**3**	Gujarat	Vadodara, Sabarkantha
**4**	Karnataka	Bangalore, Gulbarga
**5**	Madhya Pradesh	Khandwa, Gwalior
**6**	Rajasthan	Jaipur, Jhunjhunu
**7**	Tamil Nadu	Kancheepuram, Villupuram
**8**	Uttar Pradesh	Lucknow, Kanpur
**9**	West Bengal	Cooch Behar, Murshidabad
**Phase 2 (2008-2009)**		
**10**	Andhra Pradesh	Guntur, Hyderabad
**11**	Arunachal Pradesh	West Kameng, East Siang
**12**	Bihar	Patna, Munger
**13**	Goa	North Goa, South Goa
**14**	Jharkhand	Dhanbad, Jamshedpur
**15**	Maharashtra	Thane, Aurangabad
**16**	Mizoram	Aizawl, Lunglei
**17**	Nagaland	Kohima, Dimapur
**18**	Odisha	Cuttack, Khurda
**19**	Sikkim	East Sikkim, South Sikkim
**20**	Tripura	West Tripura, Dhalai District
**21**	Uttarakhand	Dehradun, Tehri Gadhwal

(Source: Ministry of Health and Family Welfare. Operational Guidelines: National Tobacco Control Programme. 2012. URL: http://www.nhmmp.gov.in/WebContent/Tobako-29102015/Operation-Guideline.pdf Accessed: July 24, 2016)

Supplementary Table S2: Socio-demographic characteristics of households included in the study (N=341,975)

Household characteristic	Households resided in a NTCP district* (n=35,581)			Households resided in a non-NTCP district (n=306,394)		
	1999-2000 (n=12,281)	2004-05 (n=13,001)	2011-12 (n=10,299)	1999-2000 (n=107,259)	2004-05 (n=110,742)	2011-12 (n=88,393)
**Sector, % [95% CI]**						
Rural	56·2 [54·6, 57·9]	54·0 [52·5, 55·6]	46·0 [43·9, 47·9]	74·3 [73·9, 74·6]	75·3 [74·9, 75·7]	71·2 [70·7, 71·7]
Urban	43·8 [42·1, 45·4]	46·0 [44·4, 47·5]	54·0 [52·0, 56·0]	25·7 [25·4, 26·0]	24·7 [24·3, 25·1]	28·8 [28·3, 29·3]
**Household size, % [95% CI]**						
Number of residents ≤ 5	68·8 [67·4, 70·1]	71·8 [70·5, 73·1]	77·0 [75·3, 78·6]	66·1 [65·7, 66·5]	68·8 [68·4, 69·2]	74·0 [73·5, 74·6]
Number of residents > 5	31·2 [29·9, 32·6]	28·2 [26·9, 29·5]	23·0 [21·4, 24·7]	33·9 [33·5, 34·3]	31·2 [30·8, 31·6]	26·0 [25·4, 26·5]
**Religion, % [95% CI]**						
Hindu	81·5 [80·4, 82·6]	81·8 [80·6, 82·8]	82·0 [80·5, 83·4]	83·5 [83·2, 83·8]	83·5 [83·2, 83·9]	83·4 [83·0, 83·8]
Muslim	14·3 [13·3, 15·3]	14·0 [13·1, 15·0]	14·1 [12·8, 15·5]	10·6 [10·3, 10·9]	11·0 [10·7, 11·2]	11·6 [11·2, 12·0]
Christian	2·6 [2·3, 3·0]	2·8 [2·3, 3·3]	2·8 [2·3, 3·2]	2·7 [2·6, 2·9]	2·4 [2·3, 2·5]	2·4 [2·3, 2·6]
Others	1·6 [1·3, 1·8]	1·4 [1·1, 1·8]	1·1 [0·9, 1·4]	3·2 [3·0, 3·3]	3·1 [3·0, 3·2]	2·6 [2·5, 2·8]
**Caste, % [95% CI]**						
Scheduled Tribe (ST)	7·7 [7·0, 8·3]	7·2 [6·5, 7·9]	8·3 [7·4, 9·3]	9·0 [8·8, 9·2]	9·0 [8·8, 9·3]	8·8 [8·5, 9·2]
Scheduled Caste (SC)	18·1 [17·0, 19·2]	19·0 [17·7, 20·3]	16·0 [14·6, 17·5]	19·3 [18·9, 19·6]	19·7 [19·4, 20·1]	19·5 [19·0, 20·0]
Other Backward Class (OBC)	24·7 [23·5, 25·9]	31·7 [30·3, 33·2]	39·3 [37·4, 41·3]	36·1 [35·7, 36·5]	41·4 [40·9, 41·8]	43·7 [43·1, 44·4]
Others	49·5 [47·9, 51·2]	42·1 [40·6, 43·6]	36·4 [34·4, 38·4]	35·6 [35·2, 36·0]	29·9 [29·5, 30·3]	28·0 [27·4, 28·5]
**Employment type (head of household), % [95% CI]**						
Self-employed	38·4 [36·9, 39·9]	42·3 [40·8, 43·8]	39·0 [37·2, 41·0]	43·7 [43·3, 44·1]	48·1 [47·6, 48·5]	46·1 [45·4, 46·7]
Regular labour	21·0 [19·5, 22·6]	21·4 [20·2, 22·7]	31·3 [29·5, 33·2]	10·4 [10·1, 10·6]	9·6 [9·4, 9·9]	17·7 [17·3, 18·2]
Casual labour	29·5 [28·1, 30·9]	24·4 [23·1, 25·7]	20·4 [18·8, 22·0]	34·1 [33·7, 34·5]	31·3 [30·8, 31·7]	28·6 [28·0, 29·2]
Others	11·1 [10·3, 11·9]	11·9 [10·8, 13·0]	9·3 [8·0, 10·7]	11·8 [11·5, 12·1]	11·0 [10·7, 11·3]	7·6 [7·2, 8·0]
**Wealth quintile, % [95% CI]**						
Poorest	19·2 [17·8, 20·7]	16·9 [15·8, 18·0]	16·9 [15·5, 18·4]	20·3 [20·0, 20·6]	20·5 [20·1, 20·9]	20·4 [19·9, 20·9]
Poor	20·2 [18·8, 21·6]	19·3 [18·1, 20·5]	20·7 [19·2, 22·3]	19·9 [19·5, 20·2]	20·1 [19·8, 20·5]	19·9 [19·4, 20·4]
Middle	20·1 [18·9, 21·4]	21·4 [20·2, 22·8]	20·2 [18·7, 21·8]	20·0 [19·6, 20·3]	19·8 [19·5, 20·2]	20·0 [19·5, 20·5]
Rich	21·0 [19·9, 22·1]	20·9 [19·7, 22·1]	21·1 [19·5, 22·7]	19·8 [19·5, 20·2]	19·9 [19·5, 20·2]	19·9 [19·3, 20·4]
Richest	19·5 [18·4, 20·7]	21·5 [20·2, 22·9]	21·1 [19·4, 23·0]	20·0 [19·7, 20·4]	19·7 [19·3, 20·0]	19·8 [19·3, 20·3]

Supplementary Table S2 continues

Proportion of household members with characteristic	Households resided in a NTCP district* (n=35,581)			Households resided in a non-NTCP district (n=306,394)		
	1999-2000 (n=12,281)	2004-05 (n=13,001)	2011-12 (n=10,299)	1999-2000 (n=107,259)	2004-05 (n=110,742)	2011-12 (n=88,393)
**Age groups, mean [95% CI]**						
0-4 years	0·09 [0·08, 0·09]	0·08 [0·07, 0·08]	0·07 [0·06, 0·07]	0·09 [0·09, 0·09]	0·09 [0·09, 0·09]	0·07 [0·07, 0·07]
5-14 years	0·20 [0·19, 0·21]	0·19 [0·18, 0·19]	0·16 [0·16, 0·17]	0·22 [0·21, 0·22]	0·20 [0·20, 0·21]	0·19 [0·18, 0·19]
15-29 years	0·28 [0·27, 0·29]	0·30 [0·29, 0·31]	0·32 [0·30, 0·33]	0·27 [0·27, 0·27]	0·27 [0·26, 0·27]	0·27 [0·27, 0·28]
30-59 year	0·35 [0·34, 0·35]	0·35 [0·34, 0·36]	0·36 [0·35, 0·37]	0·33 [0·33, 0·33]	0·35 [0·34, 0·35]	0·37 [0·37, 0·37]
≥60 years	0·08 [0·07, 0·08]	0·08 [0·08, 0·09]	0·09 [0·08, 0·09]	0·09 [0·09, 0·09]	0·09 [0·09, 0·09]	0·10 [0·10, 0·10]
**Sex, mean [95% CI]**						
Women	0·47 [0·46, 0·47]	0·47 [0·46, 0·48]	0·45 [0·44, 0·46]	0·48 [0·47, 0·48]	0·48 [0·48, 0·48]	0·48 [0·47, 0·48]
Men	0·53 [0·53, 0·54]	0·53 [0·52, 0·53]	0·55 [0·53, 0·55]	0·52 [0·52, 0·52]	0·52 [0·52, 0·52]	0·52 [0·52, 0·52]
**Educational attainment, mean [95% CI]**						
Illiterates	0·56 [0·55, 0·58]	0·75 [0·73, 0·76]	0·40 [0·38, 0·41]	0·62 [0·62, 0·63]	0·83 [0·83, 0·84]	0·49 [0·48, 0·49]
Primary	0·11 [0·10, 0·12]	0·09 [0·09, 0·10]	0·12 [0·11, 0·13]	0·11 [0·11, 0·12]	0·08 [0·07, 0·08]	0·13 [0·13, 0·13]
Middle	0·11 [0·11, 0·12]	0·07 [0·06, 0·07]	0·14 [0·13, 0·15]	0·11 [0·11, 0·11]	0·04 [0·04, 0·04]	0·14 [0·14, 0·14]
Secondary	0·09 [0·08, 0·10]	0·01 [0·01, 0·01]	0·12 [0·11, 0·13]	0·07 [0·07, 0·07]	0·01 [0·01, 0·01]	0·10 [0·10, 0·11]
Higher Secondary	0·01 [0·01, 0·01]	0·08 [0·07, 0·09]	0·10 [0·09, 0·11]	0·004 [0·004, 0·005]	0·04 [0·04, 0·04]	0·08 [0·08, 0·08]
Graduate and above	0·05 [0·05, 0·06]	-	0·12 [0·11, 0·13]	0·03 [0·03, 0·03]	-	0·06 [0·06, 0·06]

*NTCP districts are those which the National Tobacco Control Programme was implemented during 2007-08 (in 18 districts) and 2008-09 (in 24 districts).

Supplementary File 1: Difference-in-differences embedded within two-part model framework

The full specification of difference-in-differences estimators embedded within the two-part model framework is described as the following:

$$\log it(y_{ijt}) = \alpha + d_t + \beta_1 d_G + \sum_{t=2}^{3} d_t.d_G.\phi_t + \beta_2 X_{ijt} + \eta_j + \varepsilon_{it} \ldots\ldots\ldots\ldots\ldots\ldots\ldots\ldots\ldots \text{(Part 1)}$$

$$\log(V_{ijt} \mid y_{ijt} > 0) = \alpha + d_t + \gamma_1 d_G + \sum_{t=2}^{3} d_t.d_G.\lambda_t + \gamma_2 X_{ijt} + v_j + \mu_{it} \ldots\ldots\ldots\ldots\ldots\ldots \text{(Part 2)}$$

Where:

- y_{ijt} is a binary outcome variable for prevalence of bidi/cigarette consumption for household *i* living in district *j* during time period *t*;
- V_{ijt} is an outcome variable for monthly number of bidi/cigarette sticks consumed per person for household *i* living in district *j* during time period *t*;
- d_t stands for time indicators for the two later time periods (2004-05 and 2011-12) with *t* varying from 2 to 3;
- d_G is the binary indicator for households living in a NTCP when *G*=1 or non-NTCP district when *G*= 0;
- $d_{t=2}.d_G$ represents the difference-in-differences from t_1 to t_2 and between households resided in NTCP versus non-NTCP districts before the introduction of the NTCP, this is referred to as 'DID1' in the manuscript);
- $d_{t=3}.d_G$ represents the difference-in-differences from t_1 to t_3 and between households resided in NTCP versus non-NTCP districts. Only households resided in a NTCP district would have been exposed to the intervention at t_3. This is referred to as 'DID2' in the manuscript;
- X_{ijt} is the set of explanatory variables for household *i* living in district *j* during time period *t*;
- η_j and v_j are state level fixed effects; and
- ε_{it} and μ_{it} are error terms.

Supplementary Table S3: Effects of India's National Tobacco Control Programme (NTCP) on bidi & cigarette consumption based on propensity score matched intervention and control groups

**Part 1: Prevalence of households reporting bidi/cigarette consumption, adjusted odds ratio [95% CI]**		
	**Bidi n=76,709**	**Cigarette n=76,709**
**Constant**	0·50* [0·36, 0·67]	0·01* [0·01, 0·03]
**NTCP indicator**	1·14 [0·84, 1·54]	1·15 [0·86, 1·53]
**Time indicator, t_2**	0·60* [0·51, 0·71]	1.04 [0·86, 1·25]
**Time indicator, t_3**	0·68* [0·59, 0·78]	1·13 [0·88, 1·45]
**Interaction term, DID1**	1·11 [0·83, 1·50]	0·84 [0·57, 1·22]
**Interaction term, DID2**	1·11 [0·81, 1·52]	0·95 [0·62, 1·45]
**Effects attributable to the NTCP**	0·99 [0·78, 1·26]	1·13 [0·85, 1·50]

**Part 2: Log-transformed monthly consumption of bidi/cigarette sticks per person, conditional on the households reporting bidi/cigarette consumption, adjusted Coeff [95% CI]**		
	**Bidi n=15,154**	**Cigarette n=7,214**
**Constant**	3·62* [3·31, 3·93]	1·62* [1·12, 2·12]
**NTCP indicator**	-0·02 [-0·25, 0·19]	-0·27* [-0·51, -0·03]
**Time indicator, t_2**	-0·14* [-0·24, -0·04]	-0·01 [-0·14, 0·11]
**Time indicator, t_3**	-0·37* [-0·59, -0·14]	-0·48* [-0·68, -0·28]
**Interaction term, DID1**	-0·003 [-0·21, 0·20]	0·08 [-0·18, 0·34]
**Interaction term, DID2**	0·003 [-0·26, 0·26]	0·17 [-0·14, 0·49]
**Effects attributable to the NTCP**	0·006 [-0·24, 0·25]	0·09 [-0·19, 0·39]

Explanation of variables:

- NTCP indicator equals to 1 for households residing in an NTCP district, 0 otherwise;
- t_2 equals to 1 for survey year 2004-05, 0 otherwise;
- t_3 equals to 1 for survey year 2011-12, 0 otherwise;
- DID1 was the interaction between NTCP indicator and t_2;
- DID2 was the interaction between NTCP indicator and t_3;
- Effects attributable to NTCP was calculated as the difference of DID2 – DID1.

Propensity score matching was performed based on the socio-demographic characteristics of the NTCP districts. 1:1 nearest neighbour matching without replacement was applied to find for each NTCP district, a matched non-NTCP district with the most similar propensity score. Therefore the control group for this analysis included households resided in a matched non-NTCP district.

All regression models were adjusted for demographic and socioeconomic status of the households including sector (rural/urban); size (≤5/ >5 members); proportion of members in each age-group (0-4, 5-14, 15-29, 30-59, ≥60); proportion of females/males; proportion of members in each educational level (illiterate, primary, middle, secondary, higher secondary, graduate & above); religion (Hindu/Muslim/Christian/Others); caste (Scheduled Tribe/Scheduled Caste/Other Backward Class/Others); employment type (Self-employed/Regular labour/Casual labour/Others); and wealth quintile (poorest/poor/middle/rich/richest); and state level fixed effects.

CI = confidence interval. Coeff = coefficient.*Indicates p-value < 0·05.
